# Impact of COVID-19 on the Speech and Language Therapy Profession and Their Patients

**DOI:** 10.3389/fneur.2021.629190

**Published:** 2021-02-18

**Authors:** Katie Chadd, Kathryn Moyse, Pam Enderby

**Affiliations:** ^1^Research and Outcomes, Royal College of Speech and Language Therapists, London, United Kingdom; ^2^Health Services Research, The University of Sheffield, Sheffield, United Kingdom

**Keywords:** speech and language therapy, COVID-19, outcome measurement, service provision, disruption theory

## Abstract

**Introduction:** The UK's response to the COVID-19 pandemic presented multiple challenges to healthcare services including the suspension of non-urgent care. The impact on neurorehabilitation professions, including speech and language therapy (SLT), has been substantial.

**Objectives:** To review the changes to SLT services triggered by the COVID-19 pandemic with respect to referral rates, service delivery and outcomes, as well as examining the contribution of SLTs to the neurorehabilitation of COVID-19 patients.

**Methods:** Two surveys were distributed to Royal College of Speech and Language Therapists (RCSLT) members exploring experiences of service provision at 6 weeks and 22 weeks after the pandemic was declared in the UK. Responses to closed-ended questions, including questions regarding referral numbers were analyzed descriptively and compared at the two time-points. A database comprising routine clinical data from SLT services across the UK was used to compare information on patients receiving services prior to and during the pandemic. Data on COVID-19 patients was extracted, and findings are provided descriptively.

**Results:** Referrals to SLT services during the acute COVID-19 period in the UK were substantially less than in the same period in 2019. A number of service changes were common including adopting more flexible approaches to provision (such as tele-therapy) and being unable to provide services to some patients. Database analysis suggests fewer patients have accessed SLT since the pandemic began, including a reduction in neurorehabilitation patients. For those who received SLT, the outcomes did not change. SLTs supported a range of needs of COVID-19 patients. Treatment outcomes for COVID-19 patients with dysphagia were positive.

**Discussion:** The pandemic has affected neurorehabilitation and SLT services broadly: referral patterns are different, usual care has been disrupted and interventions have been modified affecting the impact on patient outcomes both positively and negatively. Some patients with COVID-19 require and benefit from SLT intervention.

## Introduction

Rehabilitation and enablement services have been modified significantly over the last decade in response to changes in demography and increasing care in the community, leading to demand outstripping capacity progressively over many years. People are living longer with complex health needs and there is increased evidence of the impact of rehabilitation services on improving independence and well-being leading to greater expectations and demand (1). Following the outbreak of severe acute respiratory syndrome coronavirus 2 (SARS-CoV-2 or COVID-19) and the subsequent global health emergency (2), neurorehabilitation services entered the response when the requirement was already outstripping supply. An important element of the multi-disciplinary approach to neurorehabilitation is speech and language therapy (SLT), which attends to the assessment and management of those with speech, language, communication and swallowing disorders. As such, this profession was similarly affected by the demand and supply contention.

The evidence indicates a large and important role of neurorehabilitation services in the response to COVID-19. It is well-documented that the virus commonly affects the functioning of the nervous system and patients sustain a degree of ill-health for several weeks post-infection (3). Common symptoms observed in post-acute COVID-19 patients include dyspnea (or shortness of breath) and muscle weakness causing mobility difficulties (4). Moreover, COVID-19 patients can experience fatigue, neuropsychological and cognitive problems, dysphagia (swallowing difficulties), and general impairments in their activities of daily living (5). Rehabilitation services, thus, are warranted and indeed critical for treating COVID-19 patients. Consequently, strains are put on non-COVID-19 related rehabilitation services, especially those occupying hospital bed spaces, as the need for re-organization arises following the increase in patient admittance (6).

By the end of February 2020, the first case of within-country transmission of COVID-19 in the UK was recorded and on March 18, 2020, UK National Health Service (NHS) providers were given the directive to postpone all non-urgent and elective activity. By March 19, 2020 many community health services were stopped. A UK-wide lockdown shortly followed on March 23, 2020, and by March 25, 2020, all NHS hospital visits were suspended, and services were told to plan for the redeployment of clinical staff, including speech and language therapists (SLTs), to attend to critical COVID-19 related services (7, 8). Individuals who were identified as being “extremely vulnerable” to catching the virus and experiencing severely ill health or death, received a governmental directive to “shield” and completely self-isolate for the lockdown period (9).

As the spread of the virus accelerated and hospitalizations surged, thus did the demand for SLTs to be part of the team managing critically ill COVID-19 patients. Dysphagia (an impairment in swallowing function) emerged as a frequent complication in such patients with estimates of around 30% of those admitted to hospital with COVID-19 needing a swallow assessment (10), and many who were intubated requiring swallow rehabilitation (11). Not only does an impairment in swallow function result in difficulties with oral feeding, but it is also a risk factor for developing aspiration pneumonia, which has also been documented in COVID-19 patients (12). However, early evidence does indicate that dysphagic COVID-19 patients can make a recovery following swallow rehabilitation (13) which in the UK is carried out by SLTs. Some questions remain as to the extent of late swallowing complications, potentially arising from virus-induced bulbar nerve damage (14) which may highlight the need for ongoing intervention. Thus, SLTs are an integral part of the intensive care unit team (15) and the longer-term rehabilitation team. Moreover, SLTs have a role in the management of dysphonia, another frequently reported symptom of the virus, reported in patients with mild to moderate COVID-19 (16). Furthermore, high rates of difficulties with vocal function following intubation has been reported (17). Thus, the pandemic has had a wide-ranging impact on SLT services arising from the suspension of many therapy services, the redeployment of clinicians, and the demand for specialists within critical, acute and rehabilitation services. Consequently, disruption to SLT services has been noted.

The theory of disruption (18) suggests that a sudden break or interruption of usual practice and break with established routines and models may lead to innovation as well as unintended consequences, both positive and negative. The Royal College of Speech and Language Therapists (RCSLT), the professional body for SLTs in the UK, was aware at an early stage that the pandemic would lead to breaks with established models of service provision. This offered the opportunity to examine the impact on service provision and patient outcomes.

There are two key ways of learning from major disruption. Firstly, being able to compare data, such as referral rates, patient characteristics, care pathways and outcomes, during a period of disruption with that recorded beforehand, is likely to give useful insight into intended and unintended consequences. The second source of information is the reactive experience of practitioners. This paper aims to utilize both methods to explore the changes to SLT practice and service delivery arising from the pandemic, specifically by asking the following two research questions:

1. How has COVID-19 impacted on SLT both generally, and in neurorehabilitation, in terms of (a) referral rates, (b) service provision and (c) therapy outcomes?
2. What is the contribution of SLT in COVID-19 management?

## Methods

A mixed methods approach was taken, including the distribution of two surveys to SLTs in the UK exploring their experiences following the outbreak of the pandemic at two different time points, and interrogation of a UK database (*The RCSLT Online Outcome Tool)*. Neither contribution to the database nor participation in the survey was mandated and were not specific to SLTs working in neurorehabilitation alone.

### Surveys

Two surveys were developed using Survey Monkey (19) and distributed to ~17,000 RCSLT members through different communication channels including newsletters and social media. The first survey was open between 23 April and 29 April 2020, inclusive, and the second ran between 12 August and 7 September 2020, inclusive. The questions for both surveys were developed iteratively by a working group consisting of SLTs and piloted by SLTs not involved in the development. They comprised open- and closed-ended questions. The analysis of and findings from the latter are reported here.

The surveys aimed to explore the experiences of UK-based SLTs by asking a series of closed-ended questions. The first survey included 15 questions, including 13 multiple choice questions, referring to the nature of changes in roles, responsibilities and duties, the extent to which intervention was being provided to individuals requiring speech and language therapy, any changes that were of benefit to clinical practice, service delivery and/or patients. The second survey included 3 questions contained in the first survey, and 46 additional questions about referral data and those developed from the often-reported experiences from the first survey, including teletherapy, workforce capacity and the barriers service users faced when accessing services. For each multiple-choice question in both surveys, participants were asked to select all statements which reflected their experience, which is analyzed descriptively regarding how often statements were selected. The full versions of both surveys can be found in the Supplementary Material.

To explore the impact of COVID-19 on SLT *referral rates* across speech and language therapy services, the responses to the questions on the second survey of “how many referrals did your service receive for speech, language and communication needs in the following periods” and “how many referrals did your service receive for dysphagia in the following periods” (periods specified as: 1 April−31 May and 1 June−31 July in 2019 and 2020) were combined, and a percentage change across the 2 years calculated. Data specifically for referrals from neurorehabilitation services was not collected.

To examine the impact on *service provision* we present findings from the surveys regarding reported experiences around changes in the roles, responsibilities and duties of SLTs, the provision of intervention and the barriers to accessing services, alongside an analysis of changes observed in the ROOT data for treatment episodes ending between 1 March 2019 and 31 August 2019 and 1 March 2020 and 31 August 2020, i.e. prior to and during the pandemic.

### The RCSLT Online Outcome Tool

The RCSLT had been supporting SLT services with routinely collecting data prior to the pandemic. The national database, the “ROOT” (20), supports SLTs from across the UK with collecting and collating data on referrals, case mix, presentation and outcomes of individuals of all ages receiving SLT. It generates reports which contributes to quality assurance and benchmarking (21). The data collected includes de-personalized patient information, including: gender, age, medical diagnosis, and descriptors on the swallowing or communication condition [using codes given in the International Statistical Classification of Diseases and Related Health Problems-10th Revision (22) herein, “ICD10 codes”], as well as information from the Therapy Outcome Measure (TOM) (23–26).

The TOM is designed to be a simple, reliable, cross-disciplinary, and cross-client group method of gathering information on the impact of enablement and rehabilitation. It has been rigorously tested for reliability and clinical validity (23–26) and comprises four domains, the first three of which are based on the WHO's International Classification of Functioning (ICF) definitions of Impairment, Activity and Participation (27). The fourth domain of well-being, of both the individual and the carer, was added to the TOM due to the finding that having an impact on well-being is an objective of most neurorehabilitation services and thus needs to be separately identified in the outcome measure. The TOM has an 11-point ordinal scale. A rating from 0 to 5 is made on each domain, where a score of 0 is profound, 3 is moderate and 5 is considered normal for the age, sex, and culture of the individual (25). A score of 0.5 or ½ a point may be used to indicate if the person is slightly better or worse than the descriptor (23–26).

The ROOT is opt-in (i.e., it is not mandatory for all SLT services to contribute to) and currently comprises data from a range of service types including NHS, independent and third-sector funded services. Timing of data entry is not regulated and is dependent on the SLTs or support staff to input information either “live” or periodically.

To examine the impact on *service provision*, the number and proportion of episodes of care from every area of SLT, and those of the 5 most frequently recorded neurological disorders (in the 2019 period) were extracted from the ROOT data and are compared with 2020 data descriptively.

To evaluate the impact of COVID-19 on *therapy outcomes*, initial and final TOM ratings were extracted for episodes of care from every area of SLT, and those of the most frequently recorded neurological disorder for the same 2019 and 2020 period as detailed earlier. Average changes in the TOM were calculated and are presented descriptively.

Finally, to inform on the *contribution of SLT in COVID-19 management*, data from the ROOT on patients who were recorded as positive for COVID-19 was extracted. This was interrogated to explore the overall numbers of patients presenting to SLT services (within the services that were contributing data), with a diagnosis of COVID-19 (by age and gender) and the focus of SLT intervention for these patients. The SLT role in neurorehabilitation of COVID-19 patients was specifically examined by analyzing the change in the ‘impairment' TOM before and after an episode of care of patients with a SLT diagnosis of dysphagia secondary to COVID-19. These are reported in categories which reflect the goal of intervention of these patients (i.e., whether the impairment is expected to improve, maintain at the same level, or if intervention is part of a managed decline). The average change in the TOM ratings was calculated and are presented descriptively.

### Ethical Considerations

This project involved use of anonymised audit data to evaluate current services as part of a service evaluation. SLTs provided minimal de-personalized data on all referred patients e.g., age, gender, diagnoses, and TOM ratings at the beginning and end of an episode of care to the ROOT database, and thorough information governance procedures were adhered to. Participants in the survey were anonymous and there were no inducements to take part.

## Results

Surveys of RCSLT members conducted in April 2020 and August-September 2020 received 544 and 413 responses, respectively. At the time of reporting, the ROOT contains data on 45,174 episodes of care from 39,534 patients, which are from 34 SLT services across the UK. Here, both sources of data are combined to answer the specific research questions.

### The Impact of COVID-19 on SLT Referral Rates

Table 1 shows the number of referrals received for speech, language and communication needs (SLCN) (from 68 SLT services) and dysphagia (from 52 SLT services) and the change in referral rate between the two time periods prior to and during the pandemic, as reported by participants of survey 2. It indicates a substantial reduction in referrals for SLCN (−31.10% change) although a relatively stable rate of dysphagia referrals (−1.29% change).

**Table 1:** Number of referrals received for speech, language and communication needs (SLCN) (from 68 SLT services) and dysphagia (from 52 SLT services).

		**Total number of referrals 1 April−31 July 2019**	**Total number of referrals 1 April−31 July 2020**	**2020 referrals expressed as a percentage of 2019 referrals (%)**	**Percentage change (%)**
**SLT need**	SLCN	10,081	6,946	68.9	−31.1
	Dysphagia	5,020	4,955	98.7	−1.3

### The Impact of COVID-19 on SLT Service Provision

95.6% of respondents (520/544) to the first survey said that the pandemic was having an impact on their professional roles, responsibilities and duties. They reported changes including use of different methods of service delivery, and a reduction in clinical caseload (referrals and serviced current caseload) being most commonly cited.

Table 2 shows several common changes to service delivery experienced by SLTs during the acute COVID-19 period (April 2020), with nearly two-thirds of respondents identifying that an *altered method of service delivery* occurred in this period (63.1%), and almost half reporting that they were no longer seeing patients directly (face-to-face) (48.9%).

**Table 2:** Frequently reported changes experienced by SLTs in April 2020, and number and percentage of respondents identifying these.

**Changes reported**	***n***	**Percentage of all respondents (%)**
Altered method of service delivery (e.g., remote delivery)	343	63.1
Reduction in routine clinical caseload	340	62.5
Reduction in referrals for patient/client groups on routine clinical caseload	278	51.1
No longer seeing patients directly	266	48.9
Restriction to the location of service delivery caused by closure of usual place of work (e.g., school, clinic)	240	44.1
Increased non-clinical tasks and/or projects	228	41.9

Table 3 explores the service provision changes in more detail but focuses on the provision for patients who *were* continuing to receive intervention in April 2020. The most commonly reported change to provision was more therapy being delivered remotely via telephone consultations (60.7%), with a high volume of respondents also citing the following changes: patients seen less frequently (44.5%), more video consultations (43.6%), more advice given to others (41.2%), alternative delivery of care due to PPE considerations (38.2%) and providing information via leaflets (28.3%).

**Table 3:** Six commonly reported changes in service provision for patients on routine caseloads who were continuing to receive intervention in April 2020, and number and percentage of respondents identifying these.

**Change in provision**	***n***	**Percentage of all respondents (%)**
More remote provision of therapy—via telephone consultations	330	60.7
Patients seen less frequently	242	44.5
More remote provision of therapy—via video consultations	237	43.6
More advice provided to others	224	41.2
Care being delivered in a different way due to considerations about PPE	208	38.2
Providing information *via* leaflets	154	28.3

Respondents reported that a significant proportion of patients were not receiving intervention, when in normal circumstances they would, for the both the acute COVID-19 period (April 2020) and later in August-September (2020). This demonstrates a negative shift over time, in that 74.6% responded that they did have patients who should be receiving intervention but who were not in April, which increased to 83.5% in the second survey in August-September. See Table 4.

**Table 4:** Number of respondents reporting whether they had patients on their caseload who were not receiving intervention but would usually do so.

	**April 2020**		**August-September 2020**	
**Response**	***n***	**Percentage of respondents (%)**	***n***	**Percentage of respondents (%)**
Yes	406	74.6	313	83.5
No	97	17.8	53	14.1
Not applicable/no response	41	7.5	9	2.4

The barriers to providing these patients with services are given in more detail (Table 5), across the two time points. The most frequently cited barrier in April was that services could not be provided due to *national guidance or local policy* (37.3%). This was still a common issue in August-September (42.7%) but moreover, there was an additional issue that *teletherapy was not appropriate* for some of these patients in the August-September survey (53.3%).

**Table 5:** Number of respondents reporting common barriers to accessing services for patients on their caseload who were not receiving intervention.

**Barrier**	**April 2020**		**August-September 2020**		**% change**
	***n***	**Percentage of respondents (%)**	***n***	**Percentage of respondents (%)**	
Staff availability	51	9.4	69	69	18.4
No suitable venue/closure of usual place of work (e.g., school, clinic) or service	181	33.3	126	126	33.6
Closure of caseloads	84	15.4	*	*	
As a result of changes to service delivery based on national guidance or local policy (of the SLT service or another setting/service)	203	37.3	160	160	42.7
Limited access to correct type of PPE	30	5.5	14	14	3.7
Risks associated with aerosol generating procedures (AGP)	87	16.0	*	*	
SLT not able to provide teletherapy/service does not have access to teletherapy	42	7.7	43	43	11.5
Patients do not have access to teletherapy	115	21.1	196	196	52.3
Teletherapy was not appropriate	*	*	200	53.3	
Patients not wishing to continue with intervention at the current time	160	29.4	160	160	42.7
Patients on my caseload have been discharged with advice to re-refer if required	64	11.8	*	*	
Individual/household was shielding	*	*	91	24.3	
Families' health and well-being needs	*	*	50	13.3	
Lack of access to interpreters/bilingual co-workers	*	*	32	8.5	
Other	79	14.5	58	58	15.5
No response	144	26.5	65	65	17.3

*N.b respondents could select more than one option, therefore, the percentages do not total 100*.

* *Question not included on survey*.

The second survey sought to explore these changes in service provision in more detail, such as the use of remote consultations by the profession. Respondents estimated that, on average, 46.2% of individuals on SLT caseloads were receiving services virtually (e.g., *via* teletherapy) which had been unusual before and at an earlier stage in the pandemic.

Data from the ROOT on completed episodes of care is presented in Table 6 detailing episodes recorded for patients with any of the 5 most common neurological disorders referred for SLT in the 2019 and 2020 periods. The number of episodes is also expressed as a percentage change across the 2 years. This illustrates a distinct reduction (of 1,523) in episodes of care either recorded or entered into the ROOT in the 2020 period compared with the 2019 period.

**Table 6:** Completed episodes of care recorded in ROOT, for whole database and broken down for the 5 most common neurological disorders, in the 2019 and 2020 periods, and expressed as a percentage change across the 2 years.

	**Number of completed episodes of care**	**1 March−31 August 2019**	**1 March−31 August 2020**	**% change**
All SLT patients	All	3663	2140	-
Neurological medical condition	Stroke^*^	619	147	-
	%	16.9%	6.9%	−10.0
	Dementia^*^	255	42	-
	%	7.0%	2.0%	−5
	Motor neuron disease	104	60	-
	%	2.8%	2.8%	0.0
	Parkinson's disease	55	27	-
	%	1.5%	1.3%	−0.2
	Brain tumor^*^	23	1	-
	%	0.6%	0.0%	−0.6

* *Indicates where a group of ICD10 codes have been counted together, which refer to a general condition, for example “Stroke” includes episodes recorded as relating to: Stroke, not specified as hemorrhage or infarction, and Cerebral Infarction*.

### The Impact of COVID-19 on SLT Outcomes

Table 7 shows the mean and median change in the TOM for all the ROOT data for both time periods in 2019 and 2020, as well specifically for stroke patients. The tables indicate that outcomes were largely positive and consistent in both cohorts. Interestingly, the data suggests that stroke patients made greater progress in their therapy in 2020 than in 2019.

**Table 7:** The average change in TOM scores for all ROOT data and specifically stroke data for both time periods in 2019 and 2020.

**Time period**	***n***		**TOM domain**				
			**Impairment**	**Activity**	**Participation**	**Well-being**	**Carer well-being**
All ROOT data 2019	3,663	Average (mean) change	0.62^*^	0.79^*^	0.52^*^	0.56^*^	0.56^*^
		Average (median) change	0.00	0.50^*^	0.00	0.00	0.00
All ROOT data 2020	2,140	Average (mean) change	0.54^*^	0.66^*^	0.58^*^	0.51^*^	0.69^*^
		Average (median) change	0.00	0.50^*^	0.50^*^	0.00	0.50^*^
Stroke data 2019	619	Average (mean) change	0.54^*^	0.64^*^	0.49	0.49	0.71^*^
		Average (median) change	0.00	0.00	0.00	0.00	0.50^*^
Stroke data 2020	147	Average (mean) change	1.07^*^	1.20^*^	1.19^*^	0.71^*^	0.54^*^
		Average (median) change	1.00^*^	1.00^*^	1.00^*^	0.50^*^	0.50^*^

* *An increase of 0.5 or more on the TOM is a clinically significant change (21) and is marked with an asterisk*.

### The Contribution of SLT in COVID-19 Management

**Figure 1:**
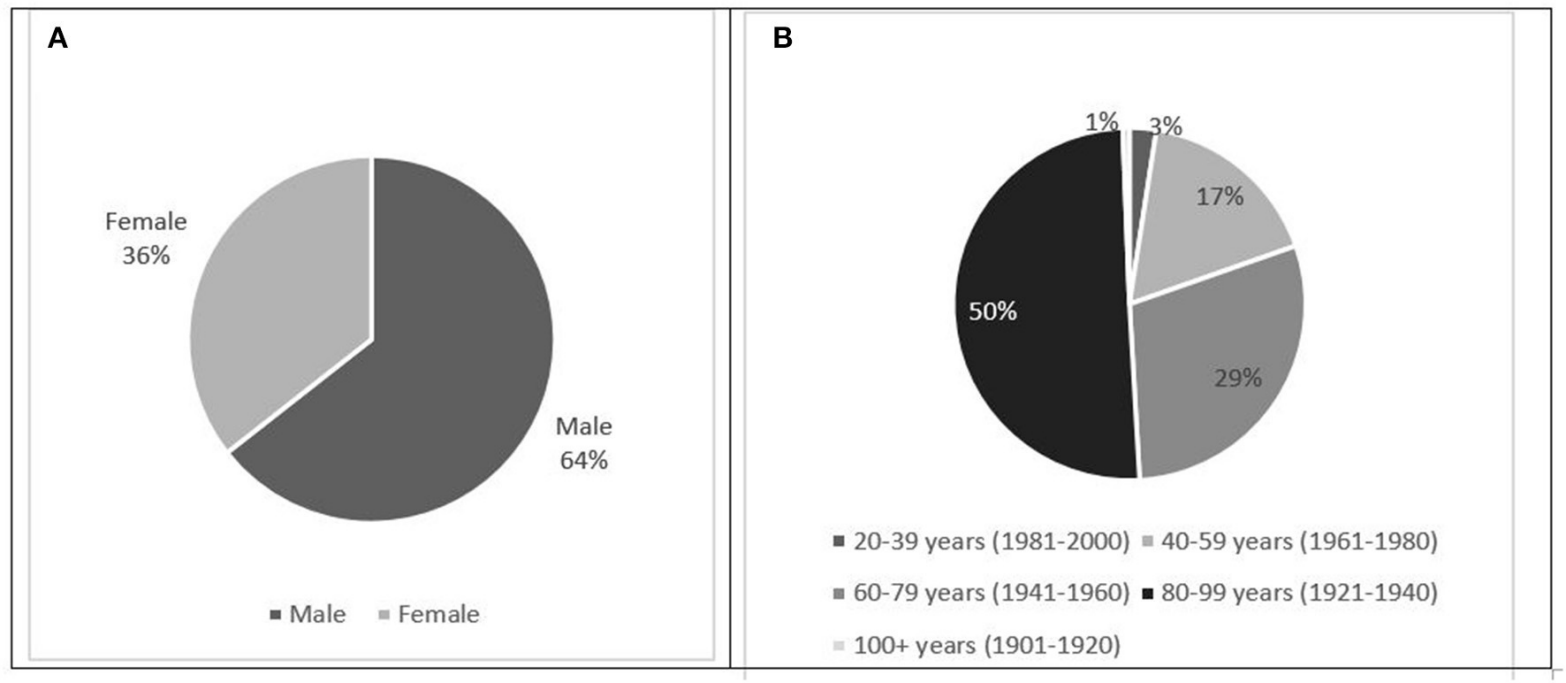
Proportion of 163 patients with confirmed COVID-19 diagnosis at the point of referral to speech and language therapy, by gender **(A)** and age group **(B)**.

The data on 163 individuals with a confirmed COVID-19 diagnosis (Figure 1) indicates that more males than females were referred, and a greater proportion of people from the older age group required SLT services, which is in line with the reported gender and severity differences related to COVID-19 requiring hospitalization (28).

The data in Table 8 describes the SLT management required for the patients referred with a positive diagnosis of COVID-19, and the average change in the TOM. These individuals were treated for dysphonia, dysphagia, dysarthria and cognitive communication disorder. Some patients were orally intubated and/or had a tracheostomy as part of their management requiring assistance with oral hygiene. This shows some variability in the degree of change for different conditions, however clinically significant changes were reported for most.

**Table 8:** The number and average change in TOM “impairment” ratings from multiple TOM scales for COVID-19 positive patients being treated by SLT services.

	**TOM scale**				
	**Dysphonia**	**Dysphagia**	**Cognitive communication disorder**	**Tracheostomy**	**Oral hygiene**
*n*	69	174	83	30	82
Average (mean) change	1.02^*^	0.83^*^	0.85^*^	3.83^*^	0.19
Average (median) change	0.00	0.25	0.00	4.00^*^	0.00

*NB. Individuals may have more than one SLT requirement*.

* *An increase of 0.5 or more on the TOM is a clinically significant change (24) and is marked with an asterisk*.

Where possible, COVID-19 patients were coded for the objective of their SLT intervention: whether their impairment was anticipated to “improve,” “sustain,” or where they may have a “managed decline,” depending on the underlying medical condition causing the speech, language, communication or swallowing disorder. Table 9 provides data on the respective average outcomes of COVID-19 patients with dysphagia, within each intervention objective. The highly significant positive change in impairment rating of those on the “improve” track may be associated with the role that SLTs have in dysphagia management of COVID-19 patients, forming a crucial part of the multi-disciplinary team (MDT) (29).

**Table 9:** The number of patients with dysphagia and COVID-19, with identified specific intervention objectives (improvement, sustain, manage decline), with the corresponding median impairment scores at the start and end of treatment, and median change over time.

**Intervention objective**	**Number of patients (*n* = 81)**	**Median impairment score (start of treatment)**	**Median impairment score (end of treatment)**	**Median change in impairment**
Improve	26	3.00	4.50	1.50^*^
Sustain	16	3.00	3.00	0.00
Managed decline	14	2.00	1.00	0.00
Not specified	25	3.50	4.00	0.00

* *An increase of 0.5 or more on the TOM is a clinically significant change (24)*.

## Discussion

Despite the challenges posed on the UK healthcare system resulting from the COVID-19 pandemic, speech and language therapists have been able to adapt their ways of working, develop specialist skills and innovate strategies to manage the consequences of a new disease. On the other hand, speech and language therapy services in the UK have, for several reasons, reduced over the acute-stage of the pandemic, and it is probable that a large proportion of patients have not received the provision they would have normally been offered. The findings we present here provide a broad insight into the ways in which this has occurred from a national perspective, which appear very much in line with reports from other UK-based allied health professions (30).

Investigating such changes, and thus assessing the impact of the pandemic, is challenging. One of the advantages of a dedicated and flexible national database, such as the ROOT, is that it provides information which can be interrogated when there is a major unanticipated disruption, such as a pandemic. This allows for analysis of the impact on services and patients exposing negative and positive effects. By comparing information gathered during the first wave of the COVID-19 crisis in the UK, with that from an identical time-period in 2019, we have been able to illustrate some of the impacts of the outbreak on usual care. The survey of professionals provides further information explaining and complementing that gathered on the database and assisting with its interpretation. Whilst we acknowledge that we cannot generalize the findings from our investigations too widely due to the opt-in and non-stratified methodologies used, it can nonetheless offer a unique perspective on UK SLT provision both before and after the onset of the COVID-19 pandemic. In so doing, we have been able to investigate specific questions posed, regarding its impact.

### Changes to Referrals to and Service Delivery of Speech and Language Therapy

Overall, the data presented here suggests that SLTs have observed substantial changes to the number of referrals to SLT, and the amount and process of therapy that they have delivered, following the UK's COVID-19 response. This is perhaps not surprising given the national restrictions and is in line with reports on the general landscape of non-COVID-19 NHS care during the pandemic (31). Indeed, it is clear from both datasets that, similar to other services (30), there has been a reduction in routine SLT caseloads. This may in part be caused by fewer new referrals for the assessment and treatment of speech, language and communication disorders. Additionally, it is likely to be an effect of SLTs being unable to provide intervention to individuals on existing caseloads following the closure of settings during lockdown, and patients opting not to access services at this time. The findings from the survey provide insight into these changes, specifically the 2019/20 year-on-year referrals, but also the finding relating to the high proportion of services which had to adopt different methods of service delivery. There has been an increase in the provision of services in different settings delivering therapy remotely using a variety of technologies, which is likely to have disadvantaged those from socially deprived areas (32) or the very elderly (33). The “switchover” to telehealth has been one of the widest reported changes to healthcare in this period (34, 35), despite its subsequent problematizing with regard to how this approach may exclude many patients without access to technology (36). These issues are likely to underpin the reduction in treatment episodes recorded on the ROOT for the pandemic.

### Impact on Neurorehabilitation Speech and Language Therapy Services

It is possible that some neurorehabilitation patient groups have been more severely affected by the pandemic, in terms of receiving therapy. We found that not only had the number of episodes of care for stroke patients reduced substantially between the 2019 and 2020 periods (619–147), the proportion of episodes of care for stroke in the 2020 period was 10% less than the year before. Whilst it is possible that over time, as more data is imported into ROOT, this pattern adjusts, it is plausible that given the COVID-19 healthcare response, these patients are simply not being referred to SLT. Early assessment and management of stroke-related dysphagia and language difficulties by SLTs reduces pneumonia and mortality (37) and there is evidence that persistent aphasia has a more favorable outcome if provided with SLTs at an early stage (38). Therefore, this finding of reduction in referrals is of concern. One explanation could be that it is simply not safe for SLTs to deliver care to these groups in the COVID-19 context (39), or these patient groups may be less able to rapidly adapt or adhere to tele-therapy (40), leading to less engagement in this period. However, the reduction observed in stroke cases from SLTs is in line with other reports showing a concerning reduction in stroke admissions across the UK throughout the lockdown period (41). Similarly, the data shows a reduction in episodes of care for dementia patients, but relatively consistent representation of patients with Motor Neuron Disease (Amyotrophic Lateral Sclerosis), Parkinson's disease and those with brain tumors. This could potentially indicate where SLT services were particularly affected, for example, with limited access to care homes to see patients with dementia, or reduced capacity in acute hospital care for those with strokes, in comparison to the likely domiciliary care for other neurorehabilitation patients with chronic or progressive diseases.

### Impact of the Pandemic on Routine Therapy Outcomes

The findings also show, interestingly and perhaps surprisingly, that the average improvement on the TOM from 2019 is indeed maintained, and in some cases, bettered, in 2020. It is clear that SLTs continue to make an impact for patients, regardless of the challenging circumstances. For the stroke patients recorded in the ROOT, the comparative average change in outcomes between 2019 and 2020 is notable; the median change in 2019 for impairment, activity and participation was 0.0, which increased to 1.0 in 2020, reflecting a positive gain of 2 half-points which is clinically significant. This is of particular interest and requires further investigation to ascertain the reasons. Yet, it is important to note that those receiving SLT in 2020 during the pandemic may be a subset of those who would do so in usual times. One consideration is that those who received intervention may have been a “less impaired” subset. It is plausible, for example, that patients with more complex needs, co-morbidities and/or those who were subject to the “shielding” regulations may have been less able to engage with services during the immediate period after the UK lockdown. Thus, this may reflect the therapy outcomes from those who were less at risk of the virus in terms of health, i.e., fewer co-morbidities and who may potentially make greater gains anyway, or those who had greater support around them from relatives/carers working at home. Another consideration may be that those from less socially deprived areas were able to access therapy more readily than those in less-affluent areas, using virtual means (32). Furthermore, some individuals may also have experienced improved access with the extension of remote delivery of services, such as those who would ordinarily find it challenging to attend appointments, due to caring responsibilities, or travel restrictions. Another explanation could be that for those engaging in teletherapy, skills acquired through intervention whilst in the home were more easily practiced and embedded than when therapy is confined to a clinic.

### Contribution of Speech and Language Therapists in Managing COVID-19

Our findings illustrate that SLT plays an important and positive role in the treatment and rehabilitation of patients with COVID-19 especially for those presenting with dysphagia, whose impairment can improve—and potentially resolve, for a subset of patients. The survey, similar to other reports (42), indicates that SLTs have adopted new roles associated with treating particular symptoms of COVID-19, such as communication with tracheostomy, and different expressions of dysphagia. The SLT profession has a growing body of data about the presentation (Table 8) and outcomes (Table 9) of individuals with COVID-19 receiving SLT intervention for the consequences of this new disease, which is further supported in the literature (12–14, 43, 44).

Going forward, it would be valuable to be able to gather information on the new ways of working from the perspective of those both receiving and in need of the service. A limitation of the study presented in this paper is the omission of information from those receiving services during this period. The RCSLT are, at the time of writing, conducting a survey to gather the experiences of individuals with speech, language, communication and swallowing needs, and their families, but the findings are not presented in this paper. Nonetheless, charities and patient associations have been collecting information on the impact of COVID-19 on their members. The impact on services was detailed in surveys conducted by the Patients' Association and the Stroke Association. The latter survey (45) received a response from 1,500 stroke survivors and carers in England, 60% of whom felt that they received less support from health and care services than was usual. Sixty-eight percent of respondents reported that they felt more anxious and depressed and more than three quarters of carers said they were providing more care and support during the pandemic than prior to it. Nine hundred and fifty-three patients responded to the survey conducted by the Patients' Association (46) which found that 67% of respondents reported that they were not seeking medical advice or intervention either because primary care services were more difficult to access or because they were avoiding contact with healthcare professionals due to anxiety related to the pandemic. These findings were not surprising given what the survey reported in this paper along with what the ROOT data indicates.

There are additional limitations to our study that should be considered when interpreting the results presented in this paper. As with all surveys, we must be cautious in our assumption that these respondents are representative of the experiences of the SLT profession within the UK. Furthermore, even though the ROOT is intended to be used by SLTs for routine data collection, it is likely that the disruption experienced by services has impacted on the ability to record outcomes data for all individuals receiving SLT compared with usual times, which may be affecting the completeness of the data in the national database. It will be important to repeat this analysis in future to explore any changes to the retrospective data. The nature of the data in the ROOT also may impede its generalizability, not least with respect to the specific UK context, but also across services within the UK, since it captures a subset of speech and language therapy services.

Despite this, we have presented an overview of the impact of COVID-19 on the role and clinical practice of SLTs in the UK, providing evidence of consequences of the pandemic, both positive and negative. The outcomes of SLT patients both prior to and during the pandemic present some interesting issues and areas for further exploration, in addition to highlighting the contribution of SLTs in COVID-19 rehabilitation. The recovery of the provision of health services once the pandemic wains will need to consider how to support those who did not receive SLT support for their speech, language, communication and swallowing needs or for their rehabilitation in a timely manner along with incorporating the new ways of working into care pathways.

## Data Availability Statement

The datasets presented in this article are not readily available because this is anonymised routine patient data. Requests to access the datasets should be directed to root@rcslt.org.

## Author Contributions

KM and KC: data collection and analysis. PE, KM, and KC: preparation of manuscript. All authors: contributed to the article and approved the submitted version.

## Conflict of Interest

The authors declare that the research was conducted in the absence of any commercial or financial relationships that could be construed as a potential conflict of interest.
