# Supplementary material for: Impact of COVID-19 on the Speech and Language Therapy Profession and Their Patients
Source: Front Neurol. 2021 Feb 18;12:629190. doi: 10.3389/fneur.2021.629190 (PMC7930219; doi:10.3389/fneur.2021.629190)
Supplement: Supplementary file 1 [file Data_Sheet_1.docx]

**Supplementary Material**

**SURVEY 1: Impact of the COVID-19 pandemic on speech and language therapy profession and provision of services**

***Introduction***

This survey has been developed by the RCSLT to explore the impact of the COVID-19 pandemic on speech and language therapy profession, the individuals it serves and provision of services.

We are interested in determining how the pandemic has:

- resulted in changes to the role of speech and language therapists
- had an impact on individual professionals
- changed the ways in which services are delivered
- had an impact on individuals on existing speech and language therapy caseloads

The RCSLT is interested in monitoring change over time, and so will consider repeating this survey in a few weeks, so it is important that you respond with information about the current, rather than expected, impact. Please respond to the following questions, based on your **experiences on the day** of completing this survey, **not** any anticipated changes.

This survey contains 15 questions and should take you less than 10 minutes to complete. The deadline for responding is Wednesday 29 April 2020.

**Question 1**

**Which clinical areas do you typically work in? (Please select all that apply)**

- AAC
- Acquired speech difficulties
- Aphasia
- Autism Spectrum Disorder
- Bilingualism
- Brain injury
- Cognitive communication disorder
- Cleft lip and palate / craniofacial
- Critical care
- Deafness
- Dementia
- Developmental language disorder
- Dysfluency
- Dyslexia
- Dysphagia (adults)
- Dysphagia (paediatrics)
- Head and neck cancer
- Learning disabilities
- Mental health (adults)
- Motor disorders
- Neonatal care
- Physical disabilities
- Progressive neurological disorders
- Public Health
- Respiratory care
- Selective mutism
- Social communication difficulties
- Social, emotional and mental health
- Speech sound disorders
- Stroke
- Tracheostomy
- Trans and gender-diverse voice and communication
- Visual and multi-sensory impairments
- Voice

**Question 2**

**Which setting(s) do you work in? (Please select all that apply)**

- Acute setting
- Care home
- Community health setting (i.e. clinics)
- Further education
- Hospice
- Hospital setting
- Mainstream school
- Nursing home
- Patient/client home
- Private practice setting
- Private school
- School – specialist unit/resource base
- Secure setting
- University
- Other (please specify)

**Question 3**

**Which RCSLT Hub region do you work in?**

- Channel Islands and Isle of Man
- East Midlands
- East of England
- London
- North East & Cumbria
- North West
- Northern Ireland
- Scotland
- South Central
- South East
- South West
- Wales
- West Midlands
- Yorkshire & the Humber

**Question 4**

The RCSLT is interested in monitoring change over time and is considering repeating this survey in a few weeks. Providing your RCSLT membership number will help us to monitor how many speech and language therapists complete the survey at more than one point in time and monitor any changes between these time points. We will not use this information to identify you and your responses will be treated anonymously.

**Your RCSLT membership number (optional):**

**Question 5**

**Is the pandemic having an impact on your professional roles, responsibilities and duties?**

- YES *(go to Q6)*
- NO *(go to Q8)*

**Question 6**

**What is the nature of the impact on your role, responsibilities and duties?**

- Expanded role due to reduction in staff availability
- Reduction in routine clinical caseload
- Reduction in referrals for patient/client groups on routine clinical caseload
- Working with different patient/client groups from routine clinical caseload
- Restriction to the location of service delivery caused by closure of usual place of work (e.g. school, clinic)
- Redeployment to support the MDT in ICU
- Redeployment to support other healthcare staff
- Redeployment to another team – physiotherapy
- Redeployment to another team – occupational therapy
- Redeployment to another team – nursing
- Redeployment to another team – other
- Redeployment to another site
- Providing support/counselling for patients/clients and their families/carers
- Altering method of service delivery (e.g. remote delivery)
- Change in duties due to being unable to attend usual place of work (e.g. due to caring commitments, myself or someone I live with being in a higher risk group (shielding/isolation))
- No longer seeing patients/clients directly
- Increased non-clinical tasks and/or projects
- Increased clinical time (e.g. returning to clinical practice)
- My research has been put on hold
- I am unable to work
- Other – please state:

**Question 7**

**What do these changes mean for you as a professional?**

- Reduced access to support /supervision/mentorship
- Lack of clarity about role
- Lacking confidence in current role
- Lacking competence in current role
- Not knowing how to help
- Absence of clinical guidelines
- Development of new care pathways
- Being a part of a new team
- Opportunities to work in new and innovative ways
- Learning new skills
- Improved MDT relationships
- Other – please state: [Free-text - limit to 15 words]

**Question 8**

**Is the pandemic having an impact on your service delivery?**

- Yes *(go to Q9)*
- No *(go to Q15)*

**Question 9**

**If patients/clients on your routine caseload are continuing to receive intervention, how has this changed? Choose the three most frequent.**

- patients/clients are seen less frequently
- care is being delivered in a different way due to considerations about PPE
- more remote provision of therapy – via telephone consultations
- more remote provision of therapy – via video consultations
- more advice provided to others
- providing information via leaflets
- patients/clients are receiving intervention from another therapist/service
- Not applicable - I am not providing intervention to any of my patients/clients on my routine caseload
- Other – please state

**Question 10**

**Are there patients/clients on your caseload who are no longer receiving intervention but would usually do so?**

- Yes
- No
- Not applicable

**Question 11**

**Why are patients/clients on your caseload no longer receiving intervention?**

- Staff availability
- Closure of usual place of work (e.g. school, clinic) or service
- Caseloads are closed
- Changes to service delivery based on national guidance or local policy*
- Limited access to correct type of PPE
- Risks associated with aerosol generating procedures (AGP)
- Our service does not have access to teletherapy
- Patients/clients do not have access to teletherapy
- Patients/clients do not wish to continue with intervention at the current time
- Patients/clients on my caseload have been discharged with advice to re-refer if required
- Other – please state:

**For example, we are aware that NHS England has published guidance* [*on COVID-19 Prioritisation within Community Health Services*](https://www.england.nhs.uk/coronavirus/wp-content/uploads/sites/52/2020/03/C0145-COVID-19-prioritisation-within-community-health-services-1-April-2020.pdf)

**Question 12:**

**Were patients/clients on your caseload informed that they are no longer receiving intervention?**

- Yes
- No
- I don’t know

**Question 13**

**Thinking about the patients/clients on your caseload who are no longer receiving intervention, what action is being taken?**

- None
- Discharged
- Placed on review
- Given advice – verbal
- Given advice – written
- Given advice – signposted to online resources
- Provided therapy in other location/by another service
- Don’t know
- Other – please state

**Question 14**

**To date, have there been any changes as a result of the pandemic that are of benefit to:**

- your clinical practice;
- your patients/clients; *and/or*
- your service

that you would like to see continue in the future?

- Yes
- No

**Question 15**

**If ‘yes’, please provide details of what changes that you would like to see continue in the future:**

Thank you for completing this survey.

The RCSLT is considering holding some focus groups at a later date to gather more detailed insights into the topics covered by this survey. If you would be interested in being involved, please provide your email address in the box below:

**SURVEY 2: RCSLT member survey: The ongoing impact of COVID-19**

***Introduction***

The RCSLT has developed a survey to explore the ongoing impact of the COVID-19 pandemic on speech and language therapy, the individuals it serves and provision of services.

**Who is it for?** The survey is aimed at all practising members who are involved in delivering or managing services across the UK.

**Purpose:** to help us understand how we can support our members, and provide evidence to influence decision makers. The survey is mostly multiple choice. Some of the questions in the survey will require you to provide data - please read this quick information sheet about the survey before completing it to ensure you have the right information to hand.

**How long will it take?**

We estimate that it will take you approximately 15-20 minutes to complete the survey. It may take service managers / heads of service / clinical leads who hold caseloads up to 30 minutes.

The deadline for responding is **5pm on Monday 7 September 2020.**

***What is the survey about?***

**Everyone** will be asked to answer questions about:

- The type of work you do - 3 questions
- The impact on individuals you support - 2 questions
- Innovations and solutions - 6 questions
- About you (diversity monitoring) - 5 questions

If you are a **service manager, head of service or clinical lead**, the survey will include questions about:

- The impact on referrals - 3 questions
- Workforce challenges and solutions - max. 12 questions

If you **hold a caseload**, the survey will include questions about:

- The impact on services and support you provide - max. 6 questions
- Use of telehealth - max. 7 questions

***About the work that you or your service does***

**Question 1**

**Which RCSLT Hub region/nation do you work in?**

- Channel Islands and Isle of Man
- East Midlands
- East of England
- London
- North East and Cumbria
- North West
- Northern Ireland
- Scotland
- South Central
- South East
- South West
- Wales
- West Midlands
- Yorkshire & the Humber

**Question 2**

**What type of work are you typically involved in?**

- Adults - acquired communication
- Adults - acquired dysphagia
- Adults with learning disabilities - communication
- Adults with learning disabilities - dysphagia
- Children and young people - communication
- Children and young people - dysphagia
- Public health (including early years)
- Other (please specify)

**Question 3**

**Which age groups do you typically work with?**

- 0-4
- 4-11
- 11-18
- 18-25
- 25-65
- 65-80
- 80+

***The impact of the pandemic on the individuals and populations you support***

**Question 4**

**Thinking about the individuals you or your service supports, since the pandemic have you observed or been informed about any of the following?**

1. **Deterioration of speech, language or communication skills**

- For many of my clients / service users
- For some of my clients / service users
- For a few of my clients / service users
- For none of my clients / service users
- Don't know
- N/A

1. **Deterioration of swallowing function**

- For many of my clients / service users
- For some of my clients / service users
- For a few of my clients / service users
- For none of my clients / service users
- Don't know
- N/A

1. **Worsening mental health**

- For many of my clients / service users
- For some of my clients / service users
- For a few of my clients / service users
- For none of my clients / service users
- Don't know
- N/A

1. **Worsening physical health**

- For many of my clients / service users
- For some of my clients / service users
- For a few of my clients / service users
- For none of my clients / service users
- Don't know
- N/A

1. **Increase in challenging behaviours**

- For many of my clients / service users
- For some of my clients / service users
- For a few of my clients / service users
- For none of my clients / service users
- Don't know
- N/A

1. **Increased barriers to accessing other services**

- For many of my clients / service users
- For some of my clients / service users
- For a few of my clients / service users
- For none of my clients / service users
- Don't know
- N/A

1. **Increase in safeguarding concerns**

- For many of my clients / service users
- For some of my clients / service users
- For a few of my clients / service users
- For none of my clients / service users
- Don't know
- N/A

**Question 5**

**Please provide more detail about the impact of the pandemic on your clients or service users, including any evidence you have to support this. This could be in the form or assessment/outcome data, or feedback from other professionals, families/carers or the clients themselves.**

**Question 6**

**Are you a service manager, head of service, clinical lead or similar? If you select 'yes' we will ask you to provide data on referrals and answer questions about workforce capacity at a service level.**

- Yes (go to Q8)
- No (go to Q7)

**Question 7**

**Do you hold your own caseload?**

- Yes (go to Q26)
- No (go to Q39)

***Impact on referrals***

**Question 8**

**What type of service are you responding on behalf of? Please select one only. If you work in more than one service, please choose one to respond on behalf of and ask a colleague to respond to others as appropriate.**

- Children - community
- Children - acute
- Children - combined community and acute service
- Children - independent practice
- Adult - community
- Adult - acute
- Adult - independent practice
- Adults with learning disabilities
- Justice - children and young people
- Justice - adult
- Mental health
- Other (please specify)

**Question 9**

**Please provide more details about your type of service if you wish**

**Question 10**

**How many referrals did your service receive for speech, language and communication needs in the following periods:**

- **1 April - 31 May 2019**
- **1 April - 31 May 2020**
- **1 June - 31 July 2019**
- **1 June - 31 July 2020**

**Question 11**

**How many referrals did your service receive for dysphagia** in the following periods:

- **1 April - 31 May 2019**
- **1 April - 31 May 2020**
- **1 June - 31 July 2019**
- **1 June - 31 July 2020**

**Question 12**

**Did these referrals/requests for support reflect the demographics and the incidence and prevalence rates for SLCN and dysphagia in the population you serve? Refer to ONS data and RCSLT clinical guidance:** [**https://www.rcslt.org/members/clinical-guidance**](https://www.rcslt.org/members/clinical-guidance)

1. **1 April - 31 May 2019**

- Yes
- No
- Don't know

1. **1 April - 31 May 2020**

- Yes
- No
- Don't know

1. **1 June - 31 July 2019**

- Yes
- No
- Don't know

1. **1 June - 31 July 2020**

- Yes
- No
- Don't know

**Question 13**

**Thinking about the workforce capacity within your service, did you operate services with a lower capacity (i.e. fewer staff) during the peak of the pandemic?**

- Yes (go to Q14)
- No - usual capacity throughout (go to Q15)
- Don't know (go to Q15)

**Question 14**

**Why did you operate with lower capacity?**

- Staff sickness
- Staff shielding
- Staff redeployed to another service
- Vacant posts
- Reduced demand
- Don’t know
- Other (please specify)

**Question 15**

**What did you do to manage workforce capacity in your service?**

- Utilisation of assistants / support workers
- Utilisation of students
- Utilisation of NQPs
- Use of SLTs deployed from another service/part of service
- Increased use of telehealth
- Offered a reduced service
- Don’t know
- Other (please specify)

**Question 16**

**What are your current workforce and service priorities?** **Please select your top three.**

- Keeping staff safe (e.g. access to adequate PPE)
- Supporting staff health and well-being
- Balancing caseloads with staff skill mix and availability
- Restarting services stopped during COVID-19
- Clinical income and contractual management
- Developing telehealth services / online resources
- Starting new services e.g. ICU MDT follow-up clinics
- Inputting to rehabilitation initiatives
- Reviewing service models for children and young people in light of current restrictions
- Reviewing service provision and ways of working to address inequalities
- How to retain recently restarted services in context of potential second surge
- Other (please specify)

***In the event of a second surge of cases in COVID-19***

**Question 17**

**In the event of a second surge of COVID-19 cases, what workforce challenges do you anticipate?**

- Staff sickness
- Staff burnout
- Overwhelming waiting lists and/or caseloads - e.g. pent up demand, demand to see new COVID-19 patients
- Lack of appropriately skilled SLTs
- Staff less willing to be redeployed to other services
- Workforce challenges in other services/settings
- Statutory demands from national education / health policy
- Pressure to redeploy staff to other parts of the service
- Clinical income and contractual management
- Access to / funding for IT and telehealth platforms
- No additional challenges
- Not applicable
- Other (please specify)

**Question 18**

**In the event of a second surge of COVID-19 cases, how would you manage workforce capacity?**

- Utilisation of assistants / support workers
- Utilisation of students
- Utilisation of NQPs
- Redeployment of SLT staff from other parts of service
- Increased training/support to carers/parents
- Increased training/support to other professionals
- Use of telehealth and online resources
- Not applicable
- Don't know
- Other (please specify)

**Question 19**

**In the event of a second surge of COVID-19 cases, how could you more effectively use students /**

**NQPs to increase capacity?**

- Making resources e.g. to support front-line staff with AAC
- Liaison with families by phone and video call
- Administrative tasks
- NQPs carrying out interventions remotely or face to face
- Troubleshooting telehealth issues
- Not applicable
- Other (please specify)

**Question 20**

**In the event of a second surge of COVID-19 cases, would your service benefit from more assistant time/generic therapy assistant post to support capacity?**

- Yes
- No
- Not sure

**Question 21**

**In the event of a second surge of COVID-19 cases, what would the barriers be to implementing more assistant time/a generic therapy assistant post?**

- Sufficient PPE to see patients face to face
- Staff available to support or train/upskill
- Current models of service
- Don’t know
- No barriers
- Other (please specify)

**Question 22**

**Have you developed any resources locally to support increasing workforce capacity?**

- Yes (go to Q23)
- No (go to Q24)
- Not sure (go to Q24)

**Question 23**

**Please provide details**

**Question 24**

**How could the RCSLT support you in managing workforce capacity in your service?**

- Disseminating good practice examples
- Publishing guidance
- Enabling connections
- Influencing national policy
- Webinars
- Don't know
- Other (please specify)

**Question 25**

**In addition to managing a service/being a clinical lead, do you have your own caseload?**

- Yes (go to Q26)
- No (go to Q39)

***Impact on the services and support you provide***

**Question 26**

**Since the outbreak of COVID-19, are there individuals on your caseload who have not received any intervention but would usually have done so?**

- Yes (go to Q27)
- No (go to Q30)
- Not applicable (go to Q30)

**Question 27**

**Please estimate what percentage of individuals on your caseload who would usually receive**

**intervention, have not received intervention**

**Question 28**

**What were the barriers to access?**

- Staff availability
- No suitable venue (usual venue closed)
- Limited access to appropriate PPE
- Individual cannot access to telehealth
- I was unable to provide telehealth
- Telehealth not appropriate
- Individuals did not wish to continue with intervention at the current time
- Individual / household was shielding
- Families’ health and wellbeing needs
- Lack of access to interpreters/ bilingual co-workers
- Lack of relationship with the individual / family / mistrust of professionals
- The policy of my service
- The policy of another setting/ service (e.g. care home, school, police station)
- I'm not sure
- Other (please specify)

**Question 29**

**What action was taken as a result?**

- None
- Discharged
- Placed on review
- Given advice - verbal / written
- Signposted to help/advice lines
- Provided therapy in other location/by another service
- Don’t know
- Other (please specify)

**Question 30**

**As a result of the pandemic, are there types of work that you are doing more of? Please tick all that apply**

- Producing accessible information
- Providing generic communication advice
- Contact with families / carers
- Collaborative working with other professionals
- Not sure
- Other (please specify)

**Question 31**

**Has the pandemic impacted on your collaborative working with other professionals / services as part of a multi-disciplinary team?**

- Positive impact
- Negative impact
- No impact

***Telehealth***

Telehealth is defined as the “delivery of health care services, where patients and providers are

separated by distance”. This can include the use of telephone, video conferencing and messaging

apps.

**Question 32**

**For what purposes have you used telehealth since the outbreak of COVID-19? Please tick all that apply**

- Communication - Assessment
- Communication - 1-2-1 intervention
- Communication - Review
- Communication - Coaching/modelling to another professional / carer
- Communication - Training to the wider workforce
- Communication - Group session
- Dysphagia - Assessment
- Dysphagia - 1-2-1 intervention
- Dysphagia - Review
- Dysphagia - Coaching/modelling to another professional / carer
- Dysphagia - Training to the wider workforce
- Dysphagia - Group session
- Case conferences e.g. statutory plan reviews
- Wellbeing checks
- Safeguarding checks
- Supporting clients' mental health
- Maintaining relationships with clients
- I have not used telehealth
- Other (please specify)

**Question 33**

**Are there any types of consultation that you would feel it was not appropriate to provide via**

**telehealth? Please tick all that apply**

- Communication - Assessment
- Communication - 1-2-1 intervention
- Communication - Review
- Communication - Coaching/modelling to another professional / carer
- Communication - Training to the wider workforce
- Communication - Group session
- Dysphagia - Assessment
- Dysphagia - 1-2-1 intervention
- Dysphagia - Review
- Dysphagia - Coaching/modelling to another professional / carer
- Dysphagia - Training to the wider workforce
- Dysphagia - Group session
- Case conferences e.g. statutory plan reviews
- Wellbeing checks
- Safeguarding checks
- Supporting clients' mental health
- Maintaining relationships with clients
- Not applicable
- Other (please specify)

**Question 34**

**Please estimate the percentage of your caseload who have received services via telehealth * since April 2020:**

**Question 35**

**Are there individuals on your caseload whose needs cannot be met by telehealth?**

- Yes (go to Q26)
- No (go to Q39)
- Not sure (go to Q39)

**Question 36**

**Please estimate the percentage of individuals on your caseload whose needs cannot be met by**

**telehealth**

**Question 37**

**What are the barriers which prevent these individuals’ needs being met by telehealth?**

- Equipment
- Internet access / data
- The nature of their communication needs
- Lack of appropriate services to meet cultural / linguistic needs
- Literacy skills / confidence
- Digital skills / confidence
- Reliance on other professionals to support access
- The policy of my service
- The policy of another service or setting (e.g. care home, school, police station)
- Lack of relationship with the individual / family / mistrust of professionals
- Don't know
- Other (please specify)

**Question 38**

**What ways have you found to overcome barriers to telehealth?**

**Question 39**

**Have you or your service developed innovative ways of meeting the needs of the individuals you work with, or the populations you serve, since the pandemic? If so, please tell us about them.**

**Question 40**

**Have you or your service developed ways of evaluating the impact of ways of working remotely to meet the needs of individuals you work with, or the populations you serve, since the pandemic? If so, please tell us about them.**

**Question 41**

**Have you developed new professional skills as a result of the pandemic?**

- Telehealth skills
- Skills with a new client group
- Skills in a new setting
- Leadership skills
- Influencing skills
- Not applicable
- Other (please specify)

**Question 42**

**Has the pandemic helped to raise the profile of speech and language therapy locally?**

- Yes
- No
- Not sure

**Question 43**

**How would you like to share the innovations and solutions you’ve developed with other services?**

- Online forum / community
- Webinar
- Social media
- Not applicable
- Other (please specify)

**Question 44**

**How would you like to learn from other services about their innovations and solutions?**

- Online forum / community
- Webinar
- Social media
- Not applicable
- Other (please specify)

***More about you***

Finally, to help us understand how SLTs’ experiences and needs may differ, please tell us about

yourself.

**Question 45**

**What is your ethnic group?**

**Question 46**

**What best describes your gender?**

- Female
- Male
- Non-binary
- Prefer not to say
- Prefer to self describe

**Question 47**

**Do you identify as trans?**

- Yes
- No
- Prefer not to say

**Question 48**

**What is your sexual orientation?**

- Bi
- Gay man
- Gay woman / lesbian
- Heterosexual / straight
- Prefer not to say
- Prefer to self describe

**Question 49**

**Do you consider yourself to be disabled?**

- Yes
- No
- Prefer not to say
